# Antioxidant and Cytoprotective Effects of (−)-Epigallocatechin-3-(3″-*O*-methyl) Gallate

**DOI:** 10.3390/ijms20163993

**Published:** 2019-08-16

**Authors:** Eunji Kim, Sang Yun Han, Kyeonghwan Hwang, Donghyun Kim, Eun-Mi Kim, Mohammad Amjad Hossain, Jong-Hoon Kim, Jae Youl Cho

**Affiliations:** 1 Department of Integrative Biotechnology, Sungkyunkwan University, Suwon 16419, Korea; 2 Daewoong Pharmaceutical Co., Yongin 17028, Korea; 3 Basic Research & Innovation vision, R&D Center, AmorePacific Corporation, Yongin 17074, Korea; 4 College of Veterinary Medicine, Chonbuk National University, Iksan 54596, Korea

**Keywords:** (−)-Epigallocatechin-3-(3″-*O*-methyl) gallate (3″Me-EGCG), antioxidant, cytoprotection, AKT1

## Abstract

Reactive oxygen species (ROS) are generated from diverse cellular processes or external sources such as chemicals, pollutants, or ultraviolet (UV) irradiation. Accumulation of radicals causes cell damage that can result in degenerative diseases. Antioxidants remove radicals by eliminating unpaired electrons from other molecules. In skin health, antioxidants are essential to protect cells from the environment and prevent skin aging. (−)-Epigallocatechin-3-(3″-*O*-methyl) gallate (3″Me-EGCG) has been found in limited oolong teas or green teas with distinctive methylated form, but its precise activities have not been fully elucidated. In this study, we examined the antioxidant roles of 3″Me-EGCG in keratinocytes (HaCaT cells). 3″Me-EGCG showed scavenging effects in cell and cell-free systems. Under H_2_O_2_ exposure, 3″Me-EGCG recovered cell viability and increased the expression of heme oxygenase 1 (HO-1). Under ultraviolet B (UVB) and sodium nitroprusside (SNP) exposure, 3″Me-EGCG protected keratinocytes and regulated the survival protein AKT1. By regulating the AKT1/NF-κB pathway, 3″Me-EGCG augmented cell survival and proliferation in HaCaT cells. These results indicate that 3″Me-EGCG exhibits antioxidant properties, resulting in cytoprotection against various external stimuli. In conclusion, our findings suggest that 3″Me-EGCG can be used as an ingredient of cosmetic products or health supplements.

## 1. Introduction

Free radicals are produced from various cellular processes and external sources including pollution, smoking, and ultraviolet (UV) radiation [1,2]. In cell metabolism, removal of oxygen molecules (reactive oxygen species, ROS) from respiration, lipid metabolism, or aerobic metabolism is not complete [3,4]. ROS has been described as a double-edged sword: low or moderate concentrations of ROS are helpful to the body by regulating cellular responses and immune functions, but large amounts of ROS cause oxidative stress, which can result in aging or chronic and degenerative disorders such as cancer and inflammatory diseases [3,4,5]. For these reasons, removal of excess ROS and maintenance of moderate concentrations of ROS are essential for human health.

**Figure 1:**
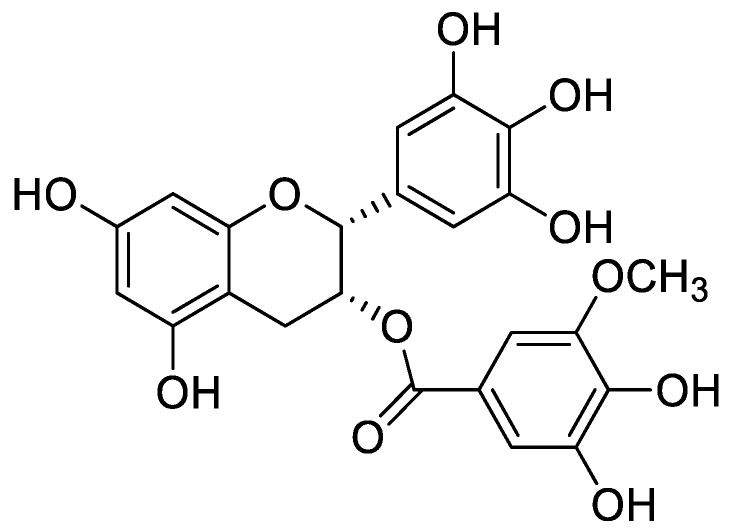
Structure of (−)-epigallocatechin-3-(3″-*O*-methyl) gallate (3″Me-EGCG).

Ultraviolet B (UVB) can damage skin cells and triggers generation of ROS [6]. Radicals induce oxidative stress and can cause cell death [7,8,9]. H_2_O_2_ (a non-radical) is generated by several oxidase enzymes such as superoxide dismutase (SOD) and xanthine oxidase, and can also induce oxidative stress [10,11,12]. Free radicals are able to be removed by direct scavenging, which results in protection from radical-mediated damage [13]. Antioxidants derived from natural products and external supply (from foods) eliminated radicals by reactive chain-breakdown or neutralization [3]. One representative natural source is green tea (*Camellia sinensis*, *Theaceae*), which contains many flavonoids, especially catechins and quercetin, and phenolic compounds including gallic acid (GC) and (−)-epigallocatechin gallate (EGCG) [8,14]. The radical scavenging effects of EGCG have been widely studied in various fields [14,15]. (−)-Epigallocatechin-3-(3’’-*O*-methyl) gallate (3″Me-EGCG) is a unique O-methylated form of EGCG and exists in oolong teas or green teas [16,17]. 3″Me-EGCG is also reported to be abundant in Amorepacific varieties of green tea Jangwon No.3 (Figure 1) [18], but its biological roles are not fully elucidated. We investigated the antioxidant effect of 3″Me-EGGG in various radical-induced conditions and cell-free systems. We determined the antioxidant roles of 3″Me-EGCG in keratinocytes under various stimuli (H_2_O_2_, UVB, and chemicals). Moreover, the antioxidant mechanism of 3″Me-EGCG was elucidated.

## 2. Results

### 2.1. 3″Me-EGCG Is a Potent Antioxidant

**Figure 2:**
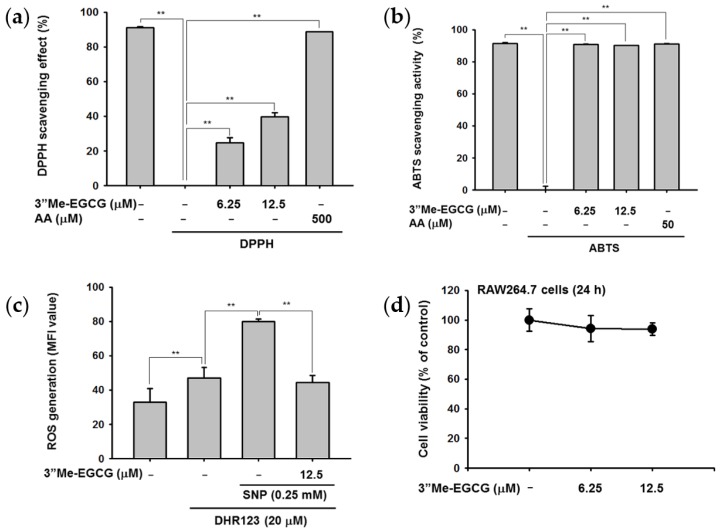
Antioxidant effects of 3″Me-EGCG. (**a**) 1-Diphenyl-2-picryl-hydrazyl (DPPH) (250 mM) was reacted with 3″Me-EGCG (0–12.5 μM) at 37 °C for 30 min. Absorbance at 417 nm was measured by spectrophotometry. Ascorbic acid was used as a positive control. (**b**) 2,2′-Azino-bis(3-ethylbenzothiazoline-6-sulphonic acid) diammonium salt (ABTS) solution was mixed with 3″Me-EGCG (0–12.5 μM) at 37 °C for 30 min. ABTS scavenging was evaluated by measuring the absorbance at 730 nm. Ascorbic acid was a positive control. (**c**) RAW264.7 cells were exposed to DHR123 for 10 min, and 3″Me-EGCG was added. After 30 min, sodium nitroprusside (SNP) was treated to induce intracellular reactive oxygen species (ROS), which was measured by flow cytometry. (**d**) The cytotoxicity of 3″Me-EGCG in RAW264.7 cells was tested by (3-4-5-Dimethylthiazol-2-yl)-2-5-diphenyltetrazolium bromide (MTT) assay. AA: ascorbic acid. ** *p* < 0.01 compared to normal (untreated) or positive (induced) group.

A previous study examined the antioxidant effects of tea leaf extract of a 3″Me-EGCG-rich tea cultivar by 1-diphenyl-2-picryl-hydrazyl (DPPH) and 2,2′-azino-bis(3-ethylbenzothiazoline-6-sulphonic acid) diammonium salt (ABTS) assay and found that the extract showed higher antioxidant activities compared with other tea cultivars [9]. We thus examined the antioxidant effects of 3″Me-EGCG. We found that 3″Me-EGCG cleared DPPH-derived radicals in a dose-dependent manner, with an IC_50_ value of 36.54 μM (Figure 2a). ABTS assay results showed that 3″Me-EGCG dramatically scavenged radicals (Figure 2b), and the IC_50_ value was 2.59 μM.

Next, it was examined whether 3″Me-EGCG could regulate intracellular ROS production in RAW264.7 cells. We found that 3″Me-EGCG was not cytotoxic to RAW264.7 cells at 0–12.5 μM (Figure 2d). RAW264.7 cells were co-treated with 3″Me-EGCG and the ROS inducer sodium nitroprusside (SNP), and production of intracellular ROS was reduced (Figure 2c). Considering with results observed in DPPH and ABTS assays (Figure 2a,b), these results indicate that 3″Me-EGCG acts as an antioxidant that scavenges chemical-derived or intracellular ROS.

### 2.2. 3″Me-EGCG Increased Heme Oxygenase 1 (HO-1) Expression under H_2_O_2_ Treatment

**Figure 3:**
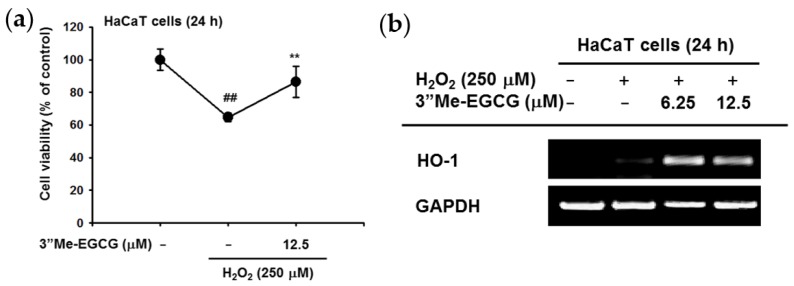
Antioxidant effects of 3″Me-EGCG against H_2_O_2_. (**a**) HaCaT cells were pre-treated with 3″Me-EGCG for 24 h, followed by H_2_O_2_. The cell viability of HaCaT cells was determined by MTT assay. (**b**) Total mRNA was prepared from 3″Me-EGCG and H_2_O_2_-treated HaCaT cells. Semi-quantitative PCR was conducted. ** *p* < 0.01 compared to normal (untreated) or positive (induced) group.

The cytoprotective effect of 3″Me-EGCG against oxidative stress conditions using H_2_O_2_ was investigated [19]. HaCaT cells were treated with 250 μM H_2_O_2_ in the absence or presence of 3″Me-EGCG, and cell viability was examined. The results showed that the cell death rate was decreased by 3″Me-EGCG (Figure 3a). The gene expression level of heme oxygenase 1 (HO-1) was quantified by semi-quantitative PCR under H_2_O_2_ conditions. The results showed that 3″Me-EGCG augmented H_2_O_2_-derived HO-1 gene expression for cytoprotection during H_2_O_2_ exposure (Figure 3b). These results show that 3″Me-EGCG could play a cytoprotective role against H_2_O_2_.

### 2.3. 3″Me-EGCG Protected Keratinocytes from SNP-Induced Radicals

**Figure 4:**
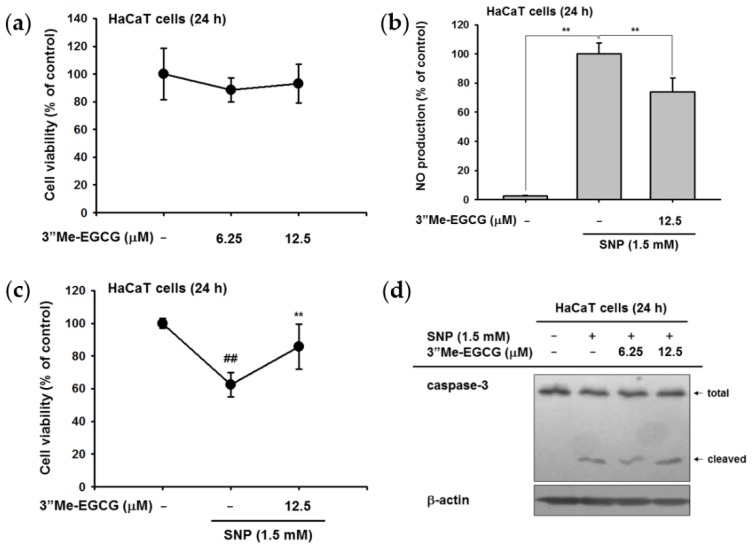
Antioxidant effects of 3″Me-EGCG against SNP-induced radicals. (**a**) The cell viability of 3″Me-EGCG-treated HaCaT cells was examined by MTT assay. (**b**) HaCaT cells were pre-treated with 3″Me-EGCG for 30 min, and then cells were exposed to SNP (1.5 mM) for 24 h. The SNP-derived nitric oxide (NO) production was determined by Griess assay. (**c**) The cell viability of SNP-treated HaCaT cells was measured in the absence or presence of 3″Me-EGCG by MTT assay. (**d**) 3″Me-EGCG- and SNP-treated HaCaT cells were lysed, and immunoblotting was performed using an antibody against caspase-3. β-actin was used as a loading control, ^##^ *p* <0.01 compared to normal group and ** *p* < 0.01 compared to normal (untreated) or positive (induced) group. −: not treated and +: treated.

Next, the potential protective role of 3″Me-EGCG under SNP treatment was examined. SNP is an ROS-inducing compound that provokes nitric oxide (NO) production and cell death [20,21]. The results showed that 3″Me-EGCG reduced SNP-derived NO production without cytotoxicity (Figure 4a,b). Simultaneously, 3″Me-EGCG protected cells from SNP-mediated cell death (Figure 4c). To dissect how 3″Me-EGCG exhibited protective effects, we performed Western blotting for the pro-apoptotic molecule caspase-3. Total and cleaved forms of caspase-3 were similar (Figure 4d), indicating that 3″Me-EGCG does not regulate the apoptotic pathway. These results implied that the cytoprotective effect of 3″Me-EGCG was not due to apoptosis regulation but to an antioxidant effect.

### 2.4. 3″Me-EGCG Protected Keratinocytes from UVB Irradiation

**Figure 5:**
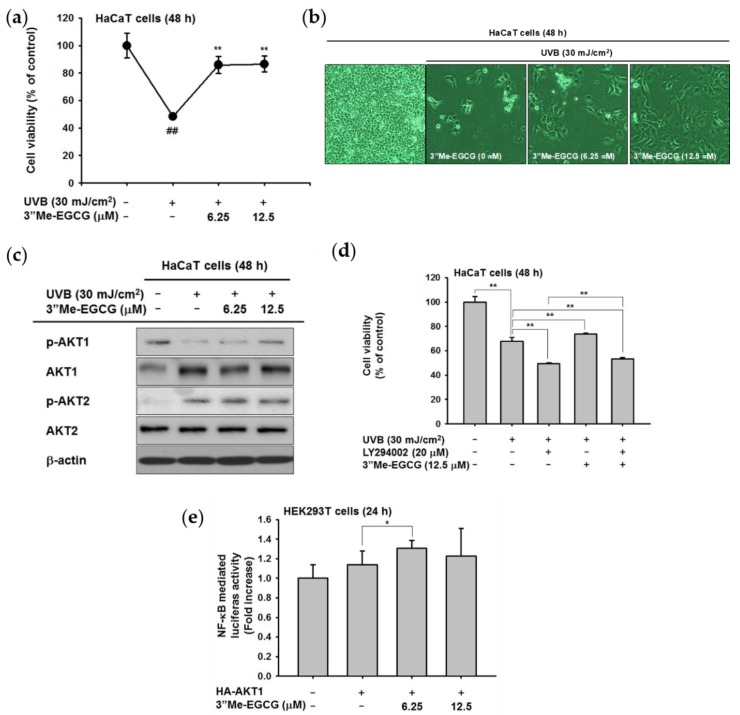
Protective effect of 3″Me-EGCG against ultraviolet B (UVB) irradiation. (**a**) HaCaT cells were pre-incubated with 3″Me-EGCG and UVB-irradiated as indicated. After 48 h, cell viability was examined by MTT assay. (**b**) Images of HaCaT cells after 3″Me-EGCG treatment and UVB irradiation. Images were captured using an optical microscope. (**c**) Whole lysate of UVB-irradiated HaCaT cells was for immunoblotting using phospho- or total antibodies against AKT1 and AKT2. β-actin was used as a loading control. (**d**) The cell viability of 3″Me-EGCG- or LY294002 (20 μM)-treated HaCaT cells in the presence of UVB was determined by MTT assay. (**e**) HA-AKT1, NF-κB-Luc, and β-galactosidase plasmids were transfected into HEK293T cells, and the cells were treated with 3″Me-EGCG (0–12.5 μM). NF-κB-mediated luciferase activity was measured by a luminometer. A β-galactosidase construct was used as a control. ^##^ *p* <0.01 compared to normal group and * *p* < 0.05 and ** *p* < 0.01 compared to normal (untreated) or positive (induced) group. -: not treated and +: treated.

Several reports have shown that antioxidants protect cells against UV irradiation [22,23,24]. The cytoprotective effect of 3″Me-EGCG against UVB irradiation was investigated. In HaCaT cells exposed to UVB (30 mJ/cm^2^), cell death was induced. However, 3″Me-EGCG protected cells from UVB, and cell viability was recovered (Figure 5a,b). Immunoblotting was used to determine the regulatory mechanism of 3″Me-EGCG against UVB-induced cell death. AKT is closely involved in cell survival [25]; thus, we determined phosphorylation levels of AKT isoforms (AKT1 and AKT2). UVB inactivated AKT1, and 3″Me-EGCG treatment resulted in restoration of AKT1 activation, while AKT2 was not affected (Figure 5c). Based on the inductive effect of 3″Me-EGCG on AKT1, the cytoprotective effect of 3″Me-EGCG was tested using an AKT inhibitor (LY294002). When AKT activity was blocked, cell viability was reduced further compared to that of UVB-irradiated HaCaT cells. However, in cells co-treated with 3″Me-EGCG and LY294002, the diminished survival rate from UVB was statistically recovered (Figure 5d). To confirm this phenomenon, a promoter assay was conducted using an NF-κB-luciferase construct. As shown in Figure 5e, AKT1-mediated NF-κB activity was elevated by 3″Me-EGCG. These data implied that AKT is correlated with the UVB-derived survival pathway, and 3″Me-EGCG protects cells by regulating AKT1.

### 2.5. 3″Me-EGCG Regulates Cell Proliferation

**Figure 6:**
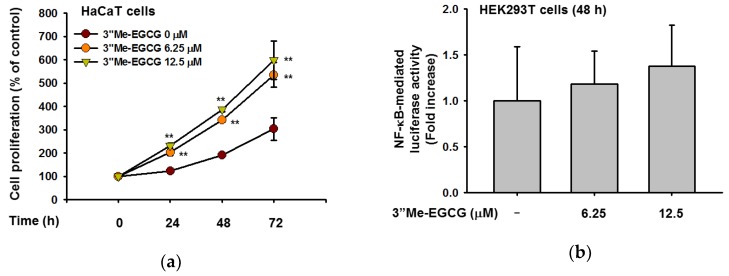
Proliferation-promoting activity of 3″Me-EGCG. (**a**) HaCaT cells (3 × 10^4^ cells/mL) were treated with 3″Me-EGCG from 0 to 72 h. At each time point, MTT solution was added, and absorbance at 570 nm was measured by spectrophotometry. (**b**) NF-κB-Luc plasmid and the control β-galactosidase construct were transfected to HEK293T cells using polyethylenimine (PEI). After 24 h, 3″Me-EGCG was added for 24 h. Luciferase activities were determined by a luminometer. ** *p* < 0.01 compared to normal (untreated) group.

Whether the cell proliferative rate of 3″Me-EGCG-treated HaCaT cells using MTT assay was determined next. For this, HaCaT cells were treated with various doses of 3″Me-EGCG for 72 h and found that 3″Me-EGCG significantly promoted cell growth at concentrations of 0–12.5 μM from 24 h (Figure 6a). To determine whether this cell proliferation-promoting activity occurs through NF-κB activation, luciferase assay was performed. Though 3″Me-EGCG was solely treated, NF-κB activities were increased by this compound (Figure 6b). These results suggest that 3″Me-EGCG triggered cell proliferation by regulating NF-κB activity.

## 3. Discussion

**Figure 7:**
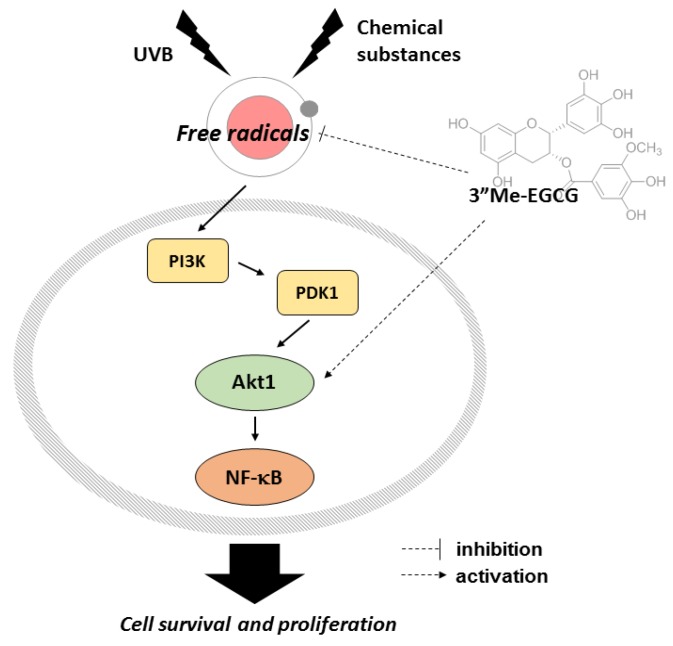
Cytoprotective and antioxidant effects of 3″Me-EGCG. 3″Me-EGCG clears free radicals from chemicals and UVB. UVB-induced free radicals regulates the AKT survival pathway, and 3″Me-EGCG regulates cell survival pathway and proliferation by targeting Akt1. 3″Me-EGCG improves cell survival with antioxidant effects.

The antioxidant effect of 3″Me-EGCG was evaluated through various analysis systems. It was revealed that 3″Me-EGCG has radical scavenging ability (Figure 2). 3″Me-EGCG protected keratinocyte cells from various stimuli including chemical substance (SNP), oxidative stress (H_2_O_2_), and UVB irradiation (Figure 3, Figure 4 and Figure 5). This cytoprotection resulted from regulation of AKT1 (Figure 5, summarized in Figure 7).

The antioxidant effect of 3″Me-EGCG was demonstrated under various radical-induced conditions—UVB (Figure 5) and chemical substances (Figure 2c and Figure 4)—and those can cause damage to skin [7,26]. Generation of radicals can also induce oxidative stress [7], so we also performed experiments using H_2_O_2_ (Figure 3). Moderate concentration of ROS is necessary to activate intracellular mechanisms, but inadequate amounts of radicals cause cell damage or death [27,28]. Generated ROS can cause not only skin aging (e.g., wrinkles and coarse texture), but also dermatological disorders including atopic dermatitis, psoriasis, and skin carcinoma [29,30,31]. Control of radicals is important to retain skin health. Our results indicate that 3″Me-EGCG can protect skin from radical-mediated damage by acting as an antioxidant.

There are two types of antioxidants: endogenous and exogenous types [4,28,32]. Endogenous antioxidants are comprised of enzymatic antioxidants (e.g., glutathione peroxidase and catalase) and non-enzymatic antioxidants (e.g., ascorbic acid and flavonoids). One of the exogenous antioxidants is a water soluble compound found in green tea [29], and 3″Me-EGCG has been found in limited amounts in green tea. For testing of exogenous antioxidant ability of 3″Me-EGCG, we investigated the cell viability with 3″Me-EGCG under SNP and UVB exposure conditions since free radicals lead to apoptosis [8,9]. Interestingly, 3″Me-EGCG protected cells from SNP (Figure 4), but cleaved caspase-3, the activated apoptotic molecule, was unaffected. These results implied that 3″Me-EGCG directly acts as an exogenous antioxidant agent. Whereas, in the case of endogenous antioxidant, HO-1 was notably affected by 3″Me-EGCG with H_2_O_2_ (Figure 3b). HO-1 serves as a cytoprotective mediator against H_2_O_2_-mediated oxidative stress [33,34,35,36], and its expression level was tested to evaluate antioxidant effects. Increase of HO-1 is essential to protect cells from oxidative stress and as an adaptive response to oxidative stress [37]. In addition, one of cell survival molecules, AKT1, was upregulated by 3″Me-EGCG in UVB-irradiated HaCaT cells (Figure 5c). Overall, these results indicate that 3″Me-EGCG functions as an endogenous and exogenous antioxidant against diverse free radical-induced conditions.

AKT (also known as protein kinase B) is a key molecule in the PI3K/AKT pathway that functions in cell proliferation and survival. Three isoforms have been described in mammalian cells, AKT1/PKBα, AKT2/PKBβ, and AKT3/PKBγ which all contain conserved domains [25,38]. AKT isoforms are highly homologous, but their expression patterns and functions are quite distinct. Knockout of AKT1 results in growth defects, whereas AKT2 deficiency leads to issues in glucose homeostasis. AKT3 knockout showed a decrease in brain size [25,39,40,41]. AKT1 and AKT3 but not AKT2 can interact with DNA-dependent protein kinase catalytic subunit (DNA-PKcs) to regulate cell proliferation [42]. AKT2 inhibited activation of c-Jun N-terminal kinase (JNK) and p38 after UVB irradiation [43]. We thus investigated which AKT isoform is essential for 3″Me-EGCG-regulated cell survival. The phosphorylated levels of AKT1 and AKT2 were opposite in pattern, and only AKT1 was affected by 3″Me-EGCG (Figure 5c). These results suggest that 3″Me-EGCG regulated cell survival by modulating AKT1 but not AKT2.

Recently, naturally-occurring antioxidants have attracted attention [26,28,36]. 3″Me-EGCG is from green tea, and our results indicate that 3″Me-EGCG exhibits antioxidative and cytoprotective effects by removal of free radicals and regulation of AKT1 and HO-1. In conclusion, our findings suggest that 3″Me-EGCG may be an ingredient applied for skin protective products as an antioxidant supplement.

## 4. Materials and Methods

### 4.1. Reagents

(−)-Epigallocatechin-3-O-(3″-*O*-methyl)-gallate (3″Me-EGCG) was purchased from Biopurify Phytocheimcals Ltd. (Chengdu, Sichuan, China) and dissolved in dimethyl sulfoxide (DMSO) to make 20 mM stock concentration. 1-Diphenyl-2-picryl-hydrazyl (DPPH), 2,2′-azino-bis(3-ethylbenzothiazoline-6-sulphonic acid) diammonium salt (ABTS), ascorbic acid, dehydrorhodamine 123 (DHR123), and sodium nitroprusside (SNP) were purchased from Sigma Chemical Co. (St. Louis, MO, USA). (3-4-5-Dimethylthiazol-2-yl)-2-5-diphenyltetrazolium bromide (MTT) was obtained from AMRESCO (Solon, OH, USA). LY294002 was from Calbiochem (La Jolla, CA, USA). Antibodies against total and phospho-AKT, -AKT1, -AKT2, and β-actin were purchased from Cell Signaling Technology (Danvers, MA, USA).

### 4.2. Cell Culture

RAW264.7 cells and HaCaT cells [American Type Culture Collection (ATCC), Manassas, VA, USA] were cultured in RPMI1640 and DMEM, respectively, with 10% FBS and 1% penicillin-streptomycin. HEK293T cells were cultured in DMEM with 5% FBS and 1% penicillin-streptomycin. All cells were incubated at 37 °C in a 5% CO_2_ humidified incubator.

### 4.3. DPPH Assay

DPPH decolorimetric assay was performed to examine the scavenging effect of 3″Me-EGCG as previously described [44,45]. 3″Me-EGCG (0–12.5 μM) was mixed with 250 mM DPPH and incubated at 37 °C for 30 min. Ascorbic acid (500 μM) was used as a positive control. After incubation, the absorbance at 517 nm was measured by spectrophotometry. DPPH scavenging effect was expressed as percent inhibition as follows:

DPPH scavenging effect (%) = [(A_0_ − A_1_)/A_0_] ∗ 100 (1)

in which A_0_ indicates the absorbance of DPPH, and A_1_ is the absorbance of samples.

### 4.4. ABTS Assay

ABTS scavenging assay was conducted as previously described with little modification [46]. A mixture of 7.4 mM ABTS and 2.4 mM potassium persulfate at a 1:1 ratio was incubated overnight at room temperature to generate ABTS radical cation (ABTS•+). ABTS solution and 3″Me-EGCG (0–12.5 μM) were mixed at a 1:1 ratio in a 96-well plate. Ascorbic acid (50 μM) was used as a positive control. After 30 min of incubation at 37°C, the absorbance of each fraction was measured at 730 nm. ABTS scavenging effect was expressed as a percentage as follows:

ABTS scavenging effect (%) = [(A_0_ − A_1_)/A_0_] ∗ 100 (2)

in which A_0_ indicates the absorbance of ABTS, and A_1_ is the absorbance of samples.

### 4.5. ROS Generation

The intracellular ROS level was determined by changes of fluorescence resulting from oxidation of the DHR123 fluorescent probe. Briefly, 1 × 10^6^ RAW264.7 cells were incubated with 3″Me-EGCG for 30 min, and then SNP (0.25 mM) was added to induce ROS production. The cells were further incubated with 20 μM of the fluorescent probe DHR123 for 30 min at 37 °C, and cells were washed with PBS. The degree of fluorescence, which corresponds to the level of intracellular ROS, was determined using a FACScan flow cytometer (Becton-Dickinson, San Jose, CA, USA) as reported previously [47,48].

### 4.6. Cell Viability Test

The cytotoxicity of 3″Me-EGCG in RAW264.7 and HaCaT cells was evaluated as previously reported [6]. RAW264.7 cells (1 × 10^6^ cells/mL) and HaCaT cells (4 × 10^6^ cells/mL) were plated and cultured overnight, and 3″Me-EGCG (0–12.5 μM) was added for 24 h. Cell culture media (100 μL) were removed, and 10 μL MTT solution was added to each well. After 3 h of formazan formation, formazan dissolving solution was added. The absorbance at 570 nm was measured.

### 4.7. UVB Irradiation

HaCaT cells were seeded at 7 × 10^5^ cells per well in six-well plates and incubated for 24 h under starvation conditions using serum-free MEM. The media were changed to DMEM with 10% FBS and 1% penicillin-streptomycin, and cells were pre-treated with 3″Me-EGCG (0–12.5 μM) for 30 min. Cells were washed with DPBS to remove media, and 1 mL media was added to each well. Cells were irradiated with 30 mJ/cm^2^ UVB (UVB lamp, Bio-link crosslinker BLX-312; Vilber Lourmat, Collegien, France). Media was removed, and DMEM media with 3″Me-EGCG (0–12.5 μM) was added to cells; cells were incubated for 48 h [49].

### 4.8. mRNA Preparation and Semi-Quantitative Polymerase Chain Reaction (PCR)

mRNA from H_2_O_2_-induced HaCaT cells was prepared to measure the expression level of HO-1. HaCaT cells were pretreated with 3″Me-EGCG for 30 min, and H_2_O_2_ (250 μM) was added for 24 h. Total RNA was isolated with TRIzol reagent following the manufacturer’s instructions. Reverse transcription PCR was conducted following the manufacturer’s instruction [50].

### 4.9. Reporter Gene Assays

HEK293T cells were seeded at 1 × 10^4^ cells per well in 24-well plates. Cells were transfected with NF-κB-Luc and β-galactosidase (control) plasmid constructs using polyethylenimine (PEI). After 24 h, media was changed, and cells were exposed to 3″Me-EGCG (0–12.5 μM) for 24 h. Luciferase activity was measured following the Luciferase Assay System (Promega; Madison, WI, USA).

### 4.10. Immunoblot Assay

Cells were washed with DPBS and then centrifuged at 12,000 rpm for 10 min at 4 °C. DPBS was removed, and the pellet was resuspended in lysis buffer (20 mM Tris-HCl pH 7.4, 2 mM EDTA, 2 mM ethylenglycoltetraacetic acid, 50 mM β-glycerophosphate, 1 mM sodium orthovanadate, 1 mM dithiotheitol, 1% Triton X-100, 10% glycerol, 10 μg/mL aprotinin, 10 μg/mL pepstatin, 1 mM benzamidine, and 2 mM PMSF). The lysates were clarified by centrifugation at 12,000 rpm for 10 min at 4 °C and stored at −20 °C until use. Protein concentrations were evaluated by Bradford assay and used for immunoblotting using antibodies against phospho- or total AKT1, AKT2, and caspase-3 by previously published methods [51]. β-actin was used as an immunoblotting loading control.

### 4.11. Cell Proliferation Assay

HaCaT cells were seeded at 3 × 10^3^ cells per well in 96-well plates and then treated with 3″Me-EGCG (0–12.5 μM) for 0–72 h. Proliferation was measured using the conventional MTT assay.

### 4.12. Giress Assay

The supernatants of cells were transferred to 96-well plates and reacted with Griess reagent (1% sulfanilamide and 0.1% N-[1-naphthyl]-ethylenediamine dihydrochloride in 5% phosphoric acid). The absorbance at 540 nm was measured by spectrophotometry [52].

### 4.13. Statistical Analysis

All data of this study are expressed as means ± standard deviations (SDs) of an experiment performed with six or two technical replicates per group. For statistical comparison, results were analyzed by ANOVA with Scheffe’s post hoc test, Kruskal–Wallis, and Mann–Whitney U tests. For all analyses, *p* < 0.05 was considered statistically significant. All statistical tests were performed with SPSS software (SPSS Inc., Chicago, IL, USA). Similar experimental data were also observed using an additional independent set of experiments that was conducted using the same numbers of samples.

## Abbreviations

3″Me-EGCG	(−)-Epigallocatechin-3-(3″-*O*-methyl) gallate
ABTS	2,2′-Azino-bis(3-ethylbenzothiazoline-6-sulphonic acid) diammonium salt
AA	Ascorbic acid
DPPH	1-Diphenyl-2-picryl-hydrazyl
EGCG	(−)-Epigallocatechin gallate
HO-1	Heme oxygenase 1
IC_50_	Half-maximal (50%) inhibitory concentration
JNK	c-Jun N-terminal kinase
MTT	(3-4-5-Dimethylthiazol-2-yl)-2-5-diphenyltetrazolium bromide
NF-κB	Nuclear factor κB
NO	Nitric oxide
PCR	Polymerase chain reaction
PKB	Protein kinase B
ROS	Reactive oxygen species
SNP	Sodium nitroprusside
UVB	Ultraviolet B
